# Methods for measuring the evolutionary stability of engineered genomes to improve their longevity

**DOI:** 10.1093/synbio/ysab018

**Published:** 2021-08-23

**Authors:** Scott L Nuismer, Nathan C. Layman, Alec J Redwood, Baca Chan, James J Bull

**Affiliations:** Department of Biological Sciences, University of Idaho, 875 Perimeter Dr, Moscow, Idaho 83844, USA; Department of Mathematics, University of Idaho, 875 Perimeter Dr, Moscow, Idaho 83844, USA; Department of Biological Sciences, University of Idaho, 875 Perimeter Dr, Moscow, Idaho 83844, USA; School of Biomedical Sciences, University of Western Australia, Perth, Western Australia, Australia; The Institute for Respiratory Health, Nedlands, Western Australia, Australia; School of Biomedical Sciences, University of Western Australia, Perth, Western Australia, Australia; The Institute for Respiratory Health, Nedlands, Western Australia, Australia; Department of Biological Sciences, University of Idaho, 875 Perimeter Dr, Moscow, Idaho 83844, USA

**Keywords:** estimation, in vitro, microbe, virus, bacteria

## Abstract

Diverse applications rely on engineering microbes to carry and express foreign transgenes. This engineered baggage rarely benefits the microbe and is thus prone to rapid evolutionary loss when the microbe is propagated. For applications where a transgene must be maintained for extended periods of growth, slowing the rate of transgene evolution is critical and can be achieved by reducing either the rate of mutation or the strength of selection. Because the benefits realized by changing these quantities will not usually be equal, it is important to know which will yield the greatest improvement to the evolutionary half-life of the engineering. Here, we provide a method for jointly estimating the mutation rate of transgene loss and the strength of selection favoring these transgene-free, revertant individuals. The method requires data from serial transfer experiments in which the frequency of engineered genomes is monitored periodically. Simple mathematical models are developed that use these estimates to predict the half-life of the engineered transgene and provide quantitative predictions for how alterations to mutation and selection will influence longevity. The estimation method and predictive tools have been implemented as an interactive web application, MuSe.

## 1. Introduction

It is now commonplace to genetically modify microbial genomes by introducing foreign genes and other elements that endow the microbe with properties of industrial, scientific or medical value but that do not benefit the individual (9, 11, 21, 28, 34, 35, 40–42). From an evolutionary perspective, such genomic additions are often selectively neutral or deleterious to individual fitness; in either case, the additions will be transient over extended periods of microbial self-propagation unless they rapidly evolve new function. Although the evolutionary durability of transgenes may be of little consequence for research that requires an intact genome for only a single assay, a short engineering half-life will be anathema to applications that require sustained transgene expression.

A common means to assess evolutionary stability of engineered genomes is to propagate the microbe in culture and monitor the genome for deletions or diminished expression (1, 8, 14, 17, 19, 25, 29, 32, 35, 39–42). In its most basic implementation, this method allows stable and highly unstable engineering to be discriminated. However, merely knowing that an engineered genome is unstable sheds little light on whether and how the stability might be improved. Unstable engineering is not irrevocable, and it can potentially be improved by further engineering or evolution (7, 14, 35, 36, 40–42).

When attempting to improve stability, it may be necessary to understand the basis of instability. Instability may be caused by a high mutation rate to engineering loss/inactivation or a high fitness cost of the engineering, and fixing one of those problems may be more important than fixing the other. Thus, when the instability was caused by a high mutation rate, changing those sequences causing the high mutation rate yielded substantial improvements in longevity (14, 21, 29, 35, 36, 40). In contrast, when the instability was tied to fitness costs associated with transgene carriage, a different construction was sometimes able to reduce the cost (1, 8, 19, 25, 35, 39, 40).

Here we develop mathematical models and statistical methodologies that allow us to estimate the mutation rate of the transgene and the fitness costs imposed on individuals that carry it. Estimating fitness effects of mutations has a long and rich history in evolutionary biology and has advanced to the level of estimating distributions of fitness effects across entire genomes (e.g., 24, 43, 45). Our approach here differs by focusing on cases common for engineered organisms where both mutation and selection can be large and evolution rapid. This falls outside the scope of traditional evolutionary biology that routinely uses approximations of weak selection and weak mutation. The models presented here are broadly similar to methods developed in a series of studies to evaluate plasmid stability (4, 5, 22, 26), but our implementations differ, and the online software we develop is specific to our methods.

## 2. Materials and Methods

The contributions of mutation and selection to evolution are intrinsically quantitative. This section develops a blend of biology and mathematics to explain our approach. The Results section provides the data analysis.

### 2.1 Modeling evolution in serial transfer experiments

**Figure 1.**
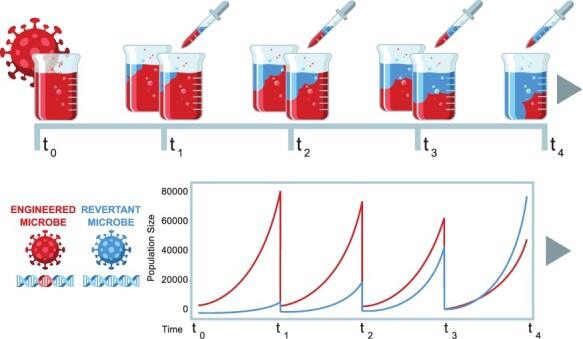
A schematic of the experimental design, genetic engineering, and evolutionary dynamics assumed by our maximum likelihood method. The top row depicts four serial transfers of a microbe, with the frequency of the engineered type (red) declining in each passage relative to the revertant microbe (blue) that has lost the engineering/insert. The lower row plots the densities of engineered and revertant strains. Microbe densities (numbers) increase over time within a culture, then are diluted precipitously at transfer, but continue growing at the same rate and eventually repopulate the new culture, only to be diluted again. The increase in frequency of the revertant strain is evident from the gradual rise to dominance of the blue curves. The transfers may continue indefinitely.

Our approach relies on a mathematical model that describes the growth of engineered and revertant strains that are propagated in culture over time using serial dilution (Figure 1). The model distinguishes selection from mutation, even though both contribute to the eventual loss of the engineering. We assume that the wild-type strain grows at a maximum per capita rate *r*, whereas the maximal per capita growth rate of the engineered strain is reduced and equal to $r(1-s)$. The parameter *s* quantifies the strength of selection acting against the foreign transgene and reflects the reduction in growth rate attributable to carrying and expressing the foreign transgene. Mutation acts as a gatekeeper of the evolutionary process, causing the engineered strain to eliminate the transgene (or its expression) with probability, *µ*, during replication. Back mutation is ignored, as transgene function, once lost, is unlikely to be regained. Once mutation has relieved an individual of its transgenic burden, selection is free to drive this more rapidly growing mutant through the population. Finally, we assume that as population size increases in culture, the growth rate of each strain decreases due to density dependence, with growth rates declining to zero as the population size approaches its carrying capacity, *K*. Together, these assumptions lead to the following system of ordinary differential equations describing the change in the population size of both types over time:

(1) \begin{align*} \frac{dN_E}{dt} &= r(1-s) N_E \left(1-\frac{N_E+N_R}{K}\right) (1-\mu)\\ \frac{dN_R}{dt} &= r N_R \left(1-\frac{N_E+N_R}{K}\right)+r (1-s)N_E \left(1-\frac{N_E+N_R}{K}\right) (\mu),\nonumber\end{align*}

with notation defined in Table 1. If there is more than one type of revertant (as regards mutation rate or fitness), an additional equation is needed for each additional type.

**Table 1.** Parameters and variables used in Equation (1)

Parameter or variable	Description
*N*_*E*_	Number of engineered individuals (function of time)
*N*_*R*_	Number of revertant individuals, equivalent to wild type (function of time)
*r*	Per capita growth rate in culture
*s*	Selection coefficient against the engineering
*K*	Carrying capacity (limit on microbial density)
*µ*	Mutation rate to loss of engineering

Before population growth ceases due to depletion of resources, serial passage experiments generally transfer a sample of culture to fresh media, with these transfers occurring repeatedly and at regular intervals (Figure 1). We model this process of repeatedly transferring culture in two ways. In the first, we assume that the amount of culture transferred is sufficiently large for stochastic effects to be negligible and evolutionary dynamics effectively deterministic; transfers occur before density-dependent effects are manifest. This approach is used to develop predictions for the half-life of the transgene and to derive a maximum likelihood estimator. In the second approach, we explicitly model the transfer process using stochastic simulations. This approach is used to simulate serial transfer experiments and allow the accuracy of our maximum likelihood method to be evaluated. Although we have motivated our model with serial passage in culture, in principle it can also be used for serial passage in multicellular organisms under some restrictions (see Discussion).

### 2.2 Predicting the lifespan of engineering

We can use a simplification of Equation (1) to predict the durability of the engineered phenotype when confronted with mutation and selection. Specifically, we calculate the time until half of the population has lost or downregulated the foreign transgene. Our approach relies on the population size remaining well below its carrying capacity, *K*. In such cases (1) can be approximated by:

(2) \begin{align*} \frac{dN_E}{dt} &= r(1-s) (1-\mu) N_E\\ \nonumber \frac{dN_R}{dt} &= r \big ( N_R + \mu(1-s)N_E \big ). \end{align*}

Applying a change of variables to these approximate equations to calculate the instantaneous rate of change for the frequency of the engineered phenotype within the population yields

(3) \begin{align*} \frac{dp}{dt} &= -rp[\mu+s(1-p-\mu)], \end{align*}

where $p = \frac{N_E}{N_E + N_R}$.

### 2.3 A maximum likelihood approach to estimating mutation and selection

The full model in (1) is too complex to solve analytically, but the approximation (3) can be solved to yield a solution for the frequency of the transgene at any time point *t* or to calculate the time until the transgene has declined to a specified value. Thus, as long as population sizes remain sufficiently large for stochastic effects to be ignored yet well below carrying capacity (i.e. small enough for the effects of density dependence to be negligible), the frequency of the transgene at time t is approximately equal to

(4) \begin{align*} p(t) =\frac{p_0\big( s(1-\mu)+\mu\big)\exp \left[-rt \big(s(1-\mu)+\mu \big)\right]} {\mu+s\left[1-\mu -p_0 \bigg(1-\exp\left(-rt \big(s(1-\mu)+\mu\big)\right)\bigg ) \right]},\end{align*}

where *p*_0_ is the frequency of the transgene at the start of the experiment. This solution for transgene frequency can be used to generate a maximum likelihood estimator for *µ* and *s* that is informed by the counts of engineered and revertant individuals in samples drawn from the culture at periodic intervals (Figure 1). Specifically, we assume that at each time *t*_*i*_ a sample is drawn from the culture, the number of engineered and revertant individuals counted, and a fresh culture is inoculated from the sample. The initial frequency *p*_0_ is calculated from a sample of individuals taken at the start of the culture, *t*_0_. It need not be 1, but as will be noted later, it is best if 1 or close to 1.

Denoting the number of individuals sampled at each time by *n* and the number of engineered individuals (carrying transgene) by *x*, the likelihood of observing the counts across the entire temporal sequence of samples is given by:

(5) \begin{equation*} L = \prod_{t\in \tau} \binom{n}{x} p(t)^x[1-p(t)]^{n-x} \end{equation*}

where *p*(*t*) is given by Equation (4) evaluated at each time *t*_*i*_ in the set of sampling times $\tau= \{t_0,t_1,{\ldots},t_J\}$. Minimizing the negative log of *L* with respect to *µ* and *s* yields the maximum likelihood estimate for these parameters given the time-series counts data. Numerical solutions are straightforward.

We evaluated the performance of our maximum likelihood estimator using simulated data. The data were generated using stochastic simulations of Equation (1). The Gillespie algorithm was used for these simulations with a version of the Tau Leaping approximation to increase speed (15; 16). The bulk of our simulations began by assuming the initial culture was pure and composed of only individuals carrying and expressing the foreign transgene. We did, however, run an additional set of simulations that relaxed this assumption by including between 5 and 50 wild-type individuals (out of 10 000) in the initial culture.

From an initial population, simulations tracked numbers within the culture using Equation (1) until the first transfer time in the experiment. At the end of this culture’s growth period, a random sample of *β* individuals was taken from the simulated culture and used to start the next culture (Figure 1). Of these *β* individuals, a subset of individuals (*n*) was analyzed to determine how many carry/express the foreign transgene (*x*). This process was repeated until the simulated experiment had run for 3600 h, 150 days. When simulating data, it is necessary to apply specific values for parameters such as growth rate, transfer interval and bottleneck size at transfer. We have focused our simulations on the scenarios and parameter combinations motivated by empirical studies with genetically engineered cytomegalovirus (Chan and Redwood, unpublished) and shown in Table 2. Experiments with bacteria, yeast or other types of virus would have different values for some or all of these parameters, but as long as the basic assumptions of the method are satisfied, the accuracy of the method should not be affected by these specifics.

**Table 2.** Model parameters and values used in simulations

Parameter	Description	Values used*
*r*	Per capita growth rate in culture	0.05, **0.1**, 0.15
*K*	Carrying capacity	2 000 000
*T*	Time between transfers	**24**, 72 (hours)
*n*	Number of individuals assayed at transfer (e.g., colonies and plaques)	25, **100**
*β*	Bottleneck size at transfer	10 000
*R*	Number of replicates	1, **3**

* If multiple values are listed, **bold** indicates the defaults

For various combinations of background parameters given in Table 2, we generated 50 simulated data sets where *µ* and *s* had been selected at random. Specifically, *s* was drawn from a uniform distribution with values ranging from 0.005 to 0.2 and *µ* was drawn at random from a Gamma distribution with mode $1.5^{\rm{R}} \times 10^{-5}$ and shape 10.0, where the mode gradually increased across the 50 simulation runs to guarantee a broad range of possible values. (The exponent ‘R’ started at 1 and was incremented by 1 with each trial, ultimately spanning values from 1 to 50.) Each of the 50 simulated data sets was then analyzed using our maximum likelihood approach, and the estimates for *µ* and *s* recorded and compared to their true values. We studied 800 such simulated data sets.

## 3. Results

### 3.1 The time to loss of engineering

To illustrate how the lifespan of the engineered phenotype depends on mutation and selection, we solved Equation (3) for the time it takes to go from a pure culture until half of the population has lost or downregulated the foreign transgene. Results derived using the Mathematica software package show that this time (*t*_50_) is

(6) \begin{align*} t_{50} = \frac{{\textrm{log}}_{\textrm{e}}\left( \frac{\mu (1-s)} {\mu (1-s) + s(1-\mu) + \mu} \right)}{-r[s(1-\mu) + \mu ]}. \end{align*}

In most cases, the goal will be to maximize this quantity, consistent with maintaining expression of the engineered phenotype for as long as possible. If there is no fitness cost to the engineering (i.e., *s* = 0), result (6) shows that *t*_50_ reduces to $0.7/(r\mu)$. Thus, if $r \mu$ is high, say 0.01, *t*_50_ will be 70 h or around 3 days. With every 10-fold decrease in mutation rate (holding *r* constant), *t*_50_ will increase 10-fold. When carrying the foreign transgene imposes a fitness cost, both mutation and selection must be quite low for *t*_50_ to exceed a couple days, except in the case that *µ* is extremely low.

**Figure 2.**
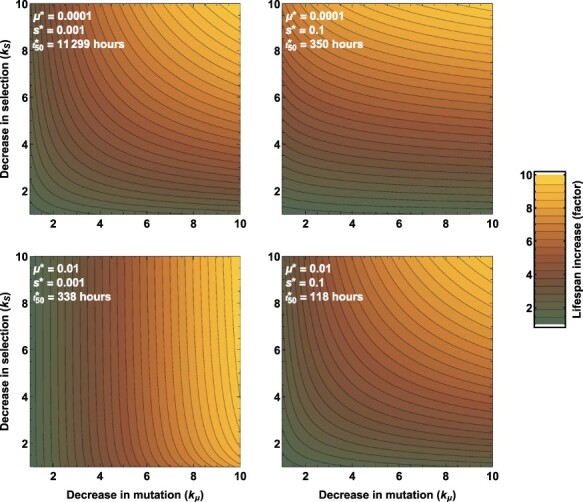
Contour plots showing how decreasing mutation rates and selection against a transgene influence the longevity of the engineering. For each panel, the starting values of mutation ($\mu^*$) and selection ($s^*$) yield the half-life value indicated (${t_{50}}^*$). The value of ${t_{50}}^*$ given in the figure applies to the lower left corner, a color value of 1 in the key at the right. As the value of mutation or selection is decreased by the factor given on the respective axis (e.g. mutation is $\mu^*/k_{\mu}$ and selection is $s^*/k_s$), the *t*_50_ value increases (the engineering persists longer) by the factor indicated in the key. Panels show a nearly 10-fold increase in longevity in the upper right corner, but the separate effects of *k*_µ_ and *k*_*s*_ vary across panels as indicated by the contour lines being vertical or horizontal.

In addition to facilitating an intuitive and quantitative understanding of how mutation and selection influence the durability of a transgene, Equation (6) provides a framework for evaluating how small changes to the engineering will influence durability. Specifically, calculating the ratio of *t*_50_ for current values of *µ* and *s* relative to the value of *t*_50_ for values of $\mu^*$ and $s^*$ that have been reduced by the factors *k*_µ_ and *k*_*s*_ respectively, clarifies when re-engineering should focus on mutation, selection, or both to maximize gains in transgene durability (Figure 2). In these plots, the initial parameters, shown in white, apply to the lower left corner of the panel, and the *t*_50_ increases as mutation and/or selection are decreased by the factor given on the respective axis. The top right panel reveals that the major effect is from decreasing selection for much of the space shown (because the isoclines are largely horizontal), whereas the lower left panel shows that the major effect is from decreasing mutation rate (because the isoclines are largely vertical).

The information provided by these estimates is only the first step in improving the half-life of the engineering. The practicality of reducing the mutation rate or selection will depend on the application. Understanding the gains from each will no doubt influence the effort expended to change the half-life.

### 3.2 Estimating mutation and selection using maximum likelihood with simulated data

Applying our maximum likelihood method to the simulated data demonstrated that the method is capable of accurately estimating both mutation and selection over a broad range of values. These simulations used Equation (1) and parameter values in Table 2; sample output data are shown in Supplementary Figure S5. There is no limit to the number of different parameter states that might be tested by simulation, but we consider the illustrations below to capture the main features of the problems investigated.

#### The estimator performs well in the ideal case, but culture duration matters.

**Figure 3.**
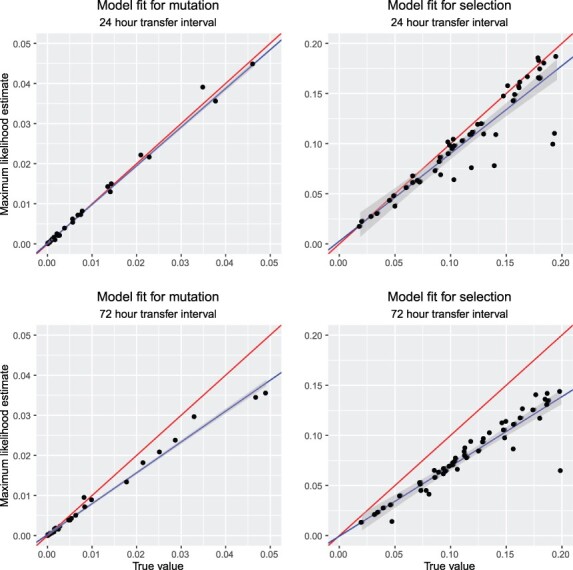
Comparisons of parameter estimates with true values using simulated data: effect of carrying capacity. The first row shows cases with transfers every 24 h, for which the population was always well below carrying capacity. On average, the estimates closely match the true values, with a best fit equation of $\mu_{estimate}=0.00017+0.96 \mu_{true}$ for mutation and $s_{estimate}=0.0027+0.87 s_{true}$ for selection. The second row analyzes cases where transfers occur every 72 h, for which the population is mostly growing non-exponentially because of its approach to carrying capacity. The estimates are now substantially biased downward, with a best fit equation of $\mu_{estimate}=0.00027+0.77 \mu_{true}$ for mutation and $s_{estimate}=-0.0005+0.70 s_{true}$ for selection. The red line indicates the 1:1 fit expected if the maximum likelihood method worked perfectly, whereas the blue line is the realized fit, with its 95% confidence interval in gray.

Estimations should perform best when the assumptions used in estimation match those used to generate the data. This outcome was found. Assuming the true *r* was known and that transfers were conducted every 24 h (hence with essentially no effect of carrying capacity), the estimated values align closely with true values (Figure 3, top row). As the transfer interval was increased to 72 h, however, the accuracy of the method fell: values of both *µ* and *s* were slightly but consistently underestimated (Figure 3, bottom row). The decrease in accuracy occurs because the 72-h population had saturated (for the value of *r* used), and its growth slowed. Reducing population growth also reduces the rate of evolution. This result emphasizes the importance of transferring the culture frequently enough for growth to remain approximately exponential.

#### Small samples can be used.

In contrast to the importance of transferring before carrying capacity is approached, our results suggest a moderately small number of individuals can be sampled at each transfer without a substantial loss in performance. Specifically, sampling only 25 individuals as opposed to 100 individuals at each transfer has no perceptible influence on the accuracy of parameter estimates when three replicates are used (Supplementary Figure S1). Likewise, the number of independent replicate transfer experiments used appears to matter little, with the precision of estimates slightly reduced for cases where only a single replicate is used with a sampling of 100 individuals at transfer (Supplementary Figure S1). The demonstration that relatively small sample sizes can be used increases the feasibility of employing our method when the state of engineering must be assayed using molecular methods rather than visually.

#### The assigned value of *r* matters.

Although the ideal scenario for application of our approach entails the independent and accurate estimation of *r*, this will not always be possible. Only ballpark estimates of *r* may be available (e.g. transfer experiments inside an organism). To evaluate the performance of our approach in such cases, we repeated the analyses of the previous paragraph with simulated data sets for which *r* was equal to either 0.05 or 0.15 but where the method assumed *r* = 0.1. The method often generated biased estimates for both *µ* and *s* (Supplementary Figure S2). In general, when the assumed *r* was larger than its true value, it underestimated the true values of *µ* and *s*. If, instead, the method assumed *r* was smaller than its true value the values of *µ* and *s* were commonly overestimates.

#### Impure initial cultures have a small effect.

Trials thus far assumed the initial culture at *t*_0_ was purely transgenic. To evaluate the importance of this assumption, simulations were performed in which the initial culture was not pure but instead included between 5 and 50 individuals lacking the transgene (a frequency of 0.0005–0.005). These numbers were chosen so that the revertant would be present initially but at a low enough frequency that it would not usually be detected in a sample of 100 individuals—the value of *n* used in these simulations. Analyses similar to those reported in the previous subsections revealed these initial impurities had only a modest impact on method performance (Supplementary Figure S3). The primary impact of starting with impure cultures was to underestimate the strength of selection. This problem can be minimized by substantially increasing the number of individuals assayed in the starting culture. If resources for conducting transfers are limiting, this result suggests it will be better to focus efforts on genotyping a larger number of individuals early in the experiment at the expense of intensive sampling later on.

#### Serial passage duration can be truncated.

Finally, we explored method accuracy when varying the duration of the serial transfer experiment. Reducing the duration of an experiment provides an obvious economy of resources, but only if accuracy is not compromised. Method performance was evaluated when serial transfer of an experiment was terminated when the frequency of the transgene reached 75%, 50% or 25% within the sample taken at transfer. Truncating replicates before the transgene is no longer detectable invariably reduces the accuracy of the estimates for mutation and selection (Supplementary Figure S4). However, provided transfers are continued until transgene frequency has reached or dipped below 25%, reductions in accuracy are slight. Earlier terminations compromise estimation.

### 3.3 An empirical application: plasmid maintenance

A simple but useful form of engineering is to add a plasmid to a bacterium. Maintenance of the plasmid is fundamentally (dynamically) the same process as maintenance of engineering in an asexual genome, where loss of the plasmid through segregation is equivalent to mutation, and faster growth of plasmid-free bacteria is equivalent to selection.

For illustration, we use part of the study of Hughes *et al.* (20): plasmids from one bacterial species were introduced into a different species and selected for maintenance with antibiotics over hundreds of generations. At various times, samples of the co-evolved cultures were propagated without the antibiotic and were then measured for plasmid absence based on whether the cell retained or lost drug resistance. The raw data were provided by E. Top (personal communication), and we applied our maximum likelihood method here to a subset of those data (estimates of mutation and selection are given in Table 3; the full data are in our Supplementary file). With cultures diluted 1000-fold every 24 h, we assumed a constant per-hour growth rate of *r* = 0.2878.

**Table 3.** Evolutionary estimates of plasmid loss parameters

Generations of coevolution	Estimated mutation rate (loss of plasmid)^1^	Estimated selection coefficient	Estimated *t*_50_ (hours)
0	0.015 (0.0078–0.023)	0.057 (0.036–0.078)	87
300	0.0009 (0.00004–0.0018)	0.040 (0.032–0.049)	325
400	0.003 (0.00045–0.0055)	0.043 (0.032–0.054)	216

1 Ranges in parentheses represent approximate 95% confidence intervals

**Figure 4.**
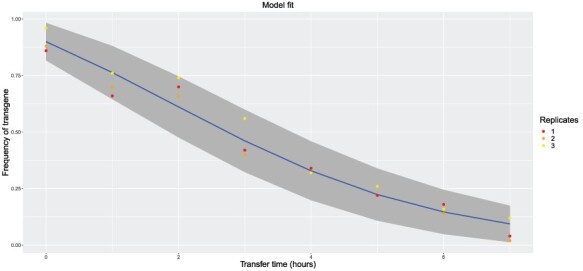
Graphical output of analysis software MuSe for the model fit to the data. Data are shown as colored dots; the blue curve is the model curve; the gray region represents the 95% confidence interval for replicate transgene frequency measurements at each time point assuming the maximum likelihood estimates are true. Data used for this figure are the Generation 0 frequencies from (20) provided by E. Top (given in our Supplementary file).

Generation 0 represents plasmid stability in the absence of any co-evolution of the plasmid with its new host. Generations 300 and 400 represent approximately 30 and 40 days of co-evolution. It is thus expected that the plasmid at the latter generations will have evolved to become more stable, either through a lower loss rate (here expressed as mutation) and/or through less costly carriage (selection). The estimates support these expectations. The estimated loss rate dropped at least 5-fold, and the cost of carriage dropped by at least 1.3-fold. The estimates for Generations 300 and 400 mostly fall within confidence limits of each other so are likely not different, but estimates of Generation 0 never lie within confidence intervals of gen-300 or gen-400 so are statistically different. The observations of changes in both plasmid stability (mutation) and fitness effect are fully compatible with the later sequence and biochemical characterizations of evolved plasmids by Yano *et al.* (44), suggesting multiple mechanisms underlying the evolutionary response. Using software described in the following section, the model fit provided for the Generation 0 data show that the data are compatible with the model-generated curve (Figure 4).

### 3.4 Software implementation

Our maximum-likelihood method is implemented in R in a web application that can be used to estimate the mutation rate and strength of selection from serial transfer experiments (http://plwa.ibest.uidaho.edu/shiny/muse); this platform was used to generate Table 3 and Figure 4. In addition to reporting the maximum likelihood estimates, this application provides plots of the likelihood surface, the fit of data simulated using the maximum likelihood estimates to the real data, and of the *t*_50_ surface predicting how changes in the mutation rate or strength of selection would influence the longevity of the engineering.

The assumptions of the growth model presented above may not always be matched by the experimental design, especially the assumption of continual exponential growth at the same rate *r*. Our software platform thus also allows an option to estimate mutation and selection on a per-culture basis (as derived in the Supplementary data). Estimates using this discrete-time model have not been validated as exhaustively as the continuous-time model, however.

## 4. Discussion

Evolution threatens the maintenance of engineered genomes by reversing or modifying the engineering over extended periods of propagation. This evolutionary challenge is greatest when the goal of the engineering requires the addition of foreign genes that provide no benefit to the parent genome or that may even be costly. Evolutionary instability results when mutation rids an individual of its transgenic cargo and selection then favors its descendants over the non-mutants. Although both mutation and selection contribute to evolutionary instability, their impacts on the half-life of genetic engineering differ, as do the methods used to adjust their magnitudes. We have developed a versatile computational method for estimating mutation and selection simultaneously using data from samples of microbial culture propagated over an extended period of serial transfers. The empirical foundation of our method is merely a standard competition assay but one in which the superior type arises through native mutation rather than by experimental addition of known mutants (6, 10, 33, 43). Our maximum likelihood estimation is suited to the simplicity of our underlying model, but the estimation could be expanded to considerable model complexity by using Approximate Bayesian Computation (ABC), albeit that the ABC approach may involve considerably more empirical analysis and computational trial and error (e.g., 2). We also acknowledge that similar but alternative approaches have been developed, specifically tailored to the problem of plasmid maintenance (4, 5, 22, 26). Our approach is broader in scope and suitable when the transgene experiences strong selection with a high mutation rate to loss.

### 4.1 What can be done with the estimates?

Understanding the magnitudes of these evolutionary processes facilitates effective and robust engineering in several ways. First, reliable estimates for the mutation rate and strength of selection allow the duration of the engineering to be predicted—whether effective for hours, days, weeks or even months. It is, of course, sometimes possible to estimate the duration of per-nucleotide empirically, but this requires potentially extended serial propagation that is practical only when engineering is short-lived. Second, estimates for mutation and selection help identify where improvements can be most easily made to increase the half-life of the engineering. For instance, finding that the rate at which mutation inactivates the transgene is marginally greater than the baseline per-nucleotide mutation rate for the genome as a whole suggests little scope for further improvement. In such a case, increasing the lifespan of the engineering must focus on reducing the selective cost of carrying the transgene, perhaps by using a functionally similar but less costly variant or by coevolving the microbe and transgene for a period of time under conditions where the transgene cannot be eliminated. In other cases, however, the finding may be the opposite and demonstrate a rate of mutation far in excess of the baseline rate but only a weak selective cost of carrying the transgene.

There are precedents for modifications to improve the longevity of engineered genomes. Sleight *et al.* (35) engineered a 2-fold increase in the evolutionary ‘half-life’ of a bacterial genetic circuit merely by removing identical terminators that had enabled recombination-mediated deletions. Similarly, the mutation rate of poliovirus was reduced by evolving the virus in a highly mutagenic environment so that its polymerase became less error-prone (37; 38). Other types of modifications may reduce fitness costs. For example, transgene stability was found to depend on the promoters expressing the transgene, presumably affecting selection based on the timing of expression (39). Kenney *et al.* (23) took an extreme approach to discovering keys to (attenuated) vaccine stability by engineering radically different vaccine designs and studying their persistence by serial transfer. There are many cases in which evolutionary instability of engineered genomes has been mitigated (see Introduction for a small sample of references), and future technical improvements in engineering platforms are only likely to make the amelioration easier.

### 4.2 Limitations

Perhaps the most serious limitation of our method is the restriction of evolution to a single mutant type. Microbial cultures commonly experience the ascent of multiple mutations, especially in the long term. When one mutation arises early and has a much larger effect than the others (e.g. a deletion of the transgene), it will dominate the estimates, an outcome that will be satisfactory for many purposes. Restricting the analysis to the early transfers may help avoid confounding secondary and tertiary mutations, but the later mutations may be the most important ones from the perspective of engineering.

Our methodology enables detecting some extreme model violations by comparing the actual data to the simulated data. Thus, if the real transgene decay data fall outside the 95% range of the simulated decay data (using the maximum likelihood estimates derived by our method), the possibility of multiple mutations must be entertained and the estimates treated with caution or abandoned. The online implementation of our method, MuSe, allows the model fit to be easily assessed in this way. Primarily because of the restriction to single mutations, we view the method developed here as a first step in the eventual development of models capable of disentangling more complex evolutionary pathways. These more sophisticated approaches could potentially integrate sequence data with phenotypic data to identify mutation rates and selection strengths for individual mutations. Whether these extensions will ever be practical for more than a couple of mutations is yet to be determined.

Our approach relies on simple models of microbial growth in culture with transfers sufficiently often to maintain exponential microbial growth. For bacteria and yeast this may be achieved by transferring culture before the population nears carrying capacity. Maintaining exponential growth of viral cultures is more difficult, however, because viral life cycles have phases in which there is no reproduction immediately after infection followed by episodic reproduction. Satisfying the model assumptions under these life histories requires transfer of a sample of the entire culture (e.g. infected cells plus free virus), timed to occur long before culture saturation (3). Constant exponential growth is not satisfied until a ‘stable age-of-infection’ distribution is attained, at which the fractions of the population in different life history stages is unchanging. Even when growth is not strictly exponential, however, our results suggest our method continues to provide reasonably good estimates for the magnitude of selection and mutation.

The estimation methods here assume that the time of transfer is known. For transfers done at regular intervals, the transfer is done based on time and use of the estimator is trivial (whether the method assumes continuous time or discrete time—the latter being addressed in the Supplementary). Some protocols may instead base transfer on the culture having reached a threshold density. Provided that the times of transfer are recorded, these latter protocols may also be analyzed with the methods developed here.

In cases where growth in culture deviates markedly from the simple exponential models used here, it may be necessary to use either more sophisticated models tuned to the biology of the specific system or to use models that ignore the within-culture details such as growth rate. Toward this end, our online software platform includes a per-transfer estimator that can be applied when the within-culture processes are unknown but consistent among cultures; no assumption about growth rate is required so long as transfer times are consistent. Estimated values of mutation and selection from the two methods (discrete and continuous time) will differ and should not be compared. Indeed, the estimates from the continuous time method depend on *r*, are per-hour, and the model assumes that all mutation and selection stops when *r* = 0; no such constraint applies to the discrete model case. However, we do expect the relative magnitudes of mutation and selection within each model to be similar across models. If the two sets of estimates are not in broad agreement, further work will be required to reconcile them.

As suggested above, modifications of our approach will be needed in the next generation of estimation models. For example, we noted that the models should accommodate multiple mutational pathways to different degrees of transgene inactivation (21, 29, 35, 40–42). Related to this is the possibility (also neglected by our model) that microbes with little prior history in culture may undergo substantial adaptive evolution, independent of the engineering. Culture adaptation leads to competing evolution that may confound our method’s ability to estimate the mutation rate and selective cost of the transgene. A simple solution to this latter problem, but not necessarily one commensurate with the engineering goals, is to engineer microbial strains already well adapted to growth in culture. Finally, a useful and perhaps easy extension of our method will be to accommodate the full model in (1) so that a carrying capacity can be applied. That extension will not lend itself to an analytical solution for *p*_*t*_ as a function of *p*_0_, rather it will likely require numerical iteration of the dynamical equations to establish that relationship.

### 4.3 Serial transfer between organisms

Although we developed our method within the context of serial passage experiments of engineered organisms in cultures, some real-world applications require growth in an organism—a plant or animal. For example, some recombinant vaccines carry a transgene and are designed to grow within the host (12, 13, 18); rapid loss of the transgene would be inimical to eliciting immunity. In this case, the relevant growth environment is the multicellular host rather than a batch of cultured cells. In principle, the same estimation methods can be applied to an engineered microbe grown in plants or animals as to growth in artificial culture. Each animal or plant host individual becomes the equivalent of a single culture. One challenge of applying our approach in this context, of course, is that the environment inside of a multicellular organism introduces considerable tissue heterogeneity that can give rise to different dynamics and evolution in different parts of the organism (27, 30, 31). An additional challenge for our continuous-time estimation lies in our model’s assumption that the selective cost of the transgene is tied to microbial growth rate. For example, when a microbe is grown in an organism, an observed growth rate (*r*) is confounded with clearance rate (equivalent to death). In the absence of modifications to our likelihood model, a microbe with both a high actual growth rate and a high clearance rate will be estimated as one with a low growth rate, in turn overestimating selection and mutation. The overall within-host process should be measured appropriately despite this error, but the combinations of $\mu, s$ and *r* will be systematically shifted from their true values. Systematic errors of this type need not generally affect the calculated *t*_50_ or fail to infer the relative contributions of mutation and selection. However, if an overestimation of the mutation rate is large, it may give an impression that attaining a lower rate is feasible when it is not.

### 4.4 Conclusions

Genetic engineering of microbial genomes is becoming increasingly routine and underpins many emerging technologies, including the development of recombinant vector vaccines and bacteria designed to deliver gene therapies. In cases where the success of the technology rests on sustained microbial replication, evolutionary instability of the engineering may undermine the objective. Presently, evolution of engineered microbial genomes appears largely idiosyncratic and difficult to generalize. By developing methods that allow the causes of evolutionary instability to be quantified, we grow closer to identifying general principles and frameworks that allow us to tune the evolutionary stability of engineered microbial genomes as well as to compete engineering techniques and approaches against each other using measurable indicators of performance.

## Supplementary Material

ysab018_Supp

## Data Availability

The methods developed in this paper have been implemented as an online resource that provides estimates from one’s own serial transfer data (http://plwa.ibest.uidaho.edu/shiny/muse). This online resource was developed in the R Shiny environment and the R script is available for download at (https://github.com/snuismer/MuSe).
