# Supplementary material for: Methods for measuring the evolutionary stability of engineered genomes to improve their longevity
Source: Synth Biol (Oxf). 2021 Aug 23;6(1):ysab018. doi: 10.1093/synbio/ysab018 (PMC8546616; doi:10.1093/synbio/ysab018)
Supplement: ysab018_Supp [file ysab018_supp.zip › supplement.pdf]

Supplemental File S1 to accompany: Methods for measuring the evolutionary stability of engineered genomes to improve their longevity

Scott L. Nuismer

Nathan Layman

Alec J. Redwood

Baca Chan

James J. Bull

1 Derivation of a discrete-time recursion

This section derives a recursion for the frequency change in individuals carrying a transgene across serial transfer. Time is discrete, and details of the dynamics within a culture are ignored. This approach will be appropriate when the process is the same between transfers but changes within a culture between the beginning and end of the culture. For example, a serial transfer process in which cultures begin in lag phase, grow exponentially for a period, and then slow at carrying capacity will not lend itself to accurate estimation for a constant r model, but selection and mutation can be estimated on a per-culture basis.

We write p_t as the frequency of the revertant at the start of culture $t \in \{0, 1, 2, ..., \Omega\}$; p_{t+1} will be the frequency at the end of culture t and equally at the start of culture $t + 1$. (The frequency of the engineered type will be $1 - p_t$, so the formula can equally be written in terms of either type.) Assuming that selection follows mutation,

$$p_1 \; = \; \frac{p_0 + \mu(1 - p_0)}{1 - s(1 - \mu)(1 - p_0)} \quad . \tag{1}$$

This equation applies to any culture as a function of frequencies in the previous culture. However, for the purposes of likelihood estimation, it is useful to have a formula for the frequency at the end of t cultures as a function of the initial frequency, p_0. Using Mathematica®, we obtain

$$p_t \; = \; \frac{\left(\frac{s}{s-1}\right)^t \left[(\mu - 1)sp_0 - \mu\right] - \mu(p_0 - 1)\left[(\mu - 1)s\right]^t}{\left(\frac{s}{s-1}\right)^t \left[(\mu - 1)sp_0 - \mu\right] - (p_0 - 1)\left[(\mu - 1)s\right]^{t+1}} \quad . \tag{2}$$

This formula has been implemented into the online software package developed for this paper (MuSe, http://plwa.ibest.uidaho.edu/shiny/muse) as an alternative approach to estimating μ and s.

2 Plasmid data

Data from the study of Hughes et al. (2012) are presented here (provided by Eva Top). A plasmid was introduced into a naive host and propagated with selection to maintain the plasmid (0 generations is the unevolved association, 300 generations is the population after 300 generations of forced co-existence, and likewise for 400 generations). At the specified generation number, a sample of the population was propagated (in 3 replicates, one transfer per day) without direct selection for plasmid maintenance, and the frequency of bacteria with and without plasmid were monitored periodically. The 24-hr cultures were saturated, so not in exponential growth at the end of the culture. At each sampling time, 50 colonies (N = 50) were plated non-selectively and assayed for plasmid presence based on drug-resistant/sensitive status. The tables below provide the frequencies of plasmid-containing cells out of 50. The last 3 lines of Table 2 were omitted from the analysis because the last two lines indicated secondary evolution – plasmid frequencies started to rise.

Table 1: Data for generation-0 association

Hours	Rep1	Rep2	Rep3	N
0	0.86	0.88	0.96	50
24	0.66	0.7	0.76	50
48	0.7	0.66	0.74	50
72	0.42	0.4	0.56	50
96	0.34	0.32	0.32	50
120	0.22	0.26	0.26	50
144	0.18	0.14	0.16	50
168	0.04	0.02	0.12	50

Table 2: Data for generation-300 association

Hours	Rep1	Rep2	Rep3	N
0	0.98	0.92	0.98	50
24	1	0.98	0.98	50
72	0.86	0.92	0.88	50
120	0.88	0.72	0.9	50
168	0.74	0.52	0.78	50
216	0.66	0.4	0.56	50
264	0.58	0.24	0.6	50
312	0.08	0.0	0.06	50
360	0.52	0.06	0.32	50
408	0.34	0.12	0.16	50

Table 3: Data for generation-400 association

Hours	Rep1	Rep2	Rep3	N
0	0.96	0.94	0.94	50
24	0.88	0.96	0.9	50
72	0.82	0.72	0.76	50
120	0.6	0.7	0.64	50
168	0.42	0.52	0.42	50
216	0.62	0.56	0.32	50
264	0.28	0.26	0.12	50
312	0.04	0.02	0.08	50

3 Figures

These figures are referenced in the text and apply to the statistical tests of the Maximum Likelihood estimator using simulated data.

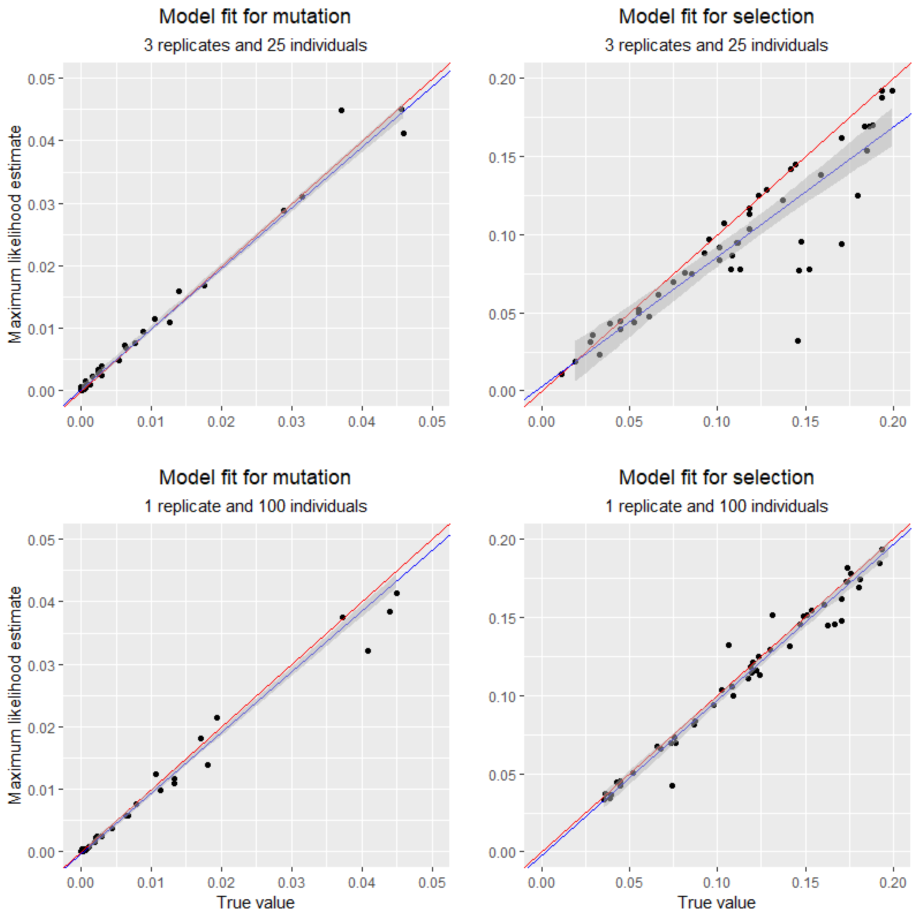

Figure S1: Comparisons of parameter estimates with true values from simulated data: effect of sample size and number of replicates. The top row shows estimates based on three replicate cultures but where only 25 individuals were tested for the presence of the transgene at each transfer. Estimates are close to true values with a best fit equation of $\mu_{estimate} = 0.0003 + 0.97\mu_{true}$ for mutation and $s_{estimate} = 0.0032 + 0.83 s_{true}$. The bottom row shows cases where only a single replicate was used and 100 individuals tested for the presence of the transgene at each transfer. Again, estimates are on average close to true values with a best fit equation of $\mu_{estimate} = -0.0002 + 0.97\mu_{true}$ for mutation and $s_{estimate} = -0.002 + 0.99 s_{true}$. The red line indicates the 1:1 fit expected if the method worked perfectly whereas the blue line indicates the realized fit. The shaded region is the 95% confidence interval for the realized fit. In all panels, transfers and sampling every 24 hours. Simulations otherwise follow those for text Fig. 3, using the bolded parameter values in Table 2.

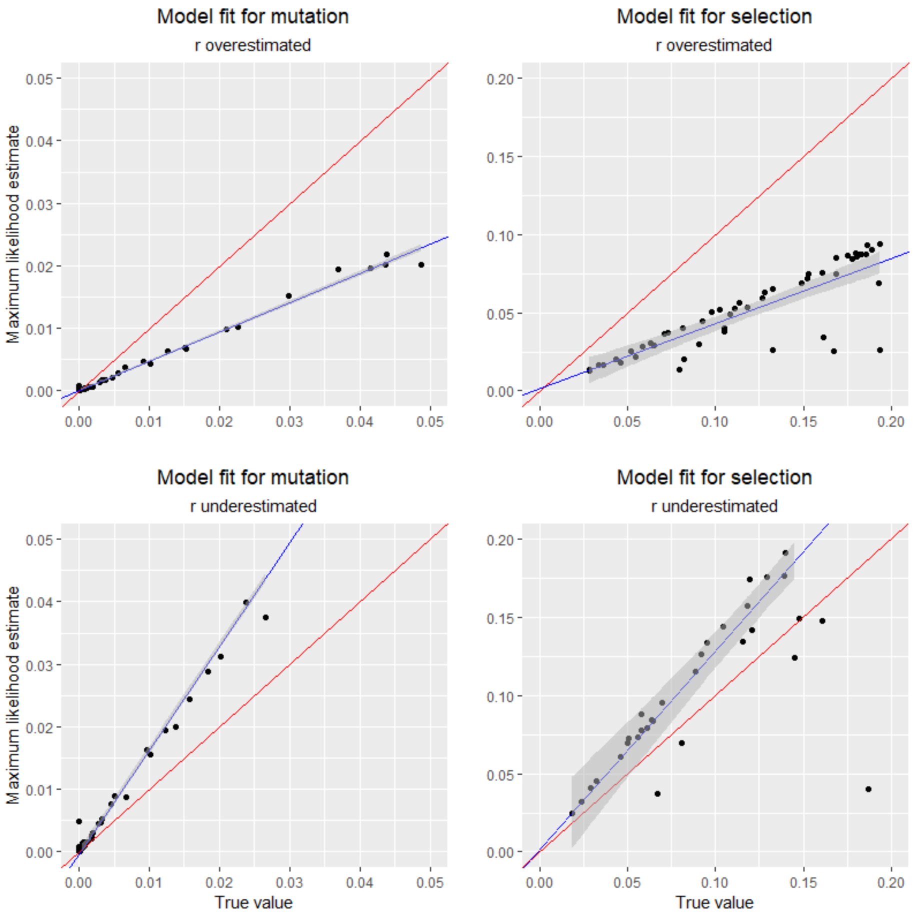

Figure S2: Comparisons of parameter estimates with true values from simulated data: effect of r. Top row: the true growth rate in culture is lower than that assumed by the method (true r = 0.05 and assumed r = 0.1). Estimates are biased downward with a best fit equation of $\mu_{estimate} = 0.0001 + 0.47\mu_{true}$ for mutation and $s_{estimate} = 0.0016 + 0.42s_{true}$. Bottom row: the true growth rate is larger than that assumed by our method (true $r = 0.15$ and assumed $r = 0.1$). Estimates are biased upward, though more so for mutation than for selection with a best fit equation of $\mu_{estimate} = -0.0002 + 1.66\mu_{true}$ for mutation and $s_{estimate} = 0.0020 + 1.27s_{true}$. The red line indicates the 1:1 fit expected if our method worked perfectly whereas the blue line indicates the realized fit. The shaded region is the 95% confidence interval for the realized fit. Simulations otherwise follow those for text Fig. 3, using the bolded parameter values in Table 2.

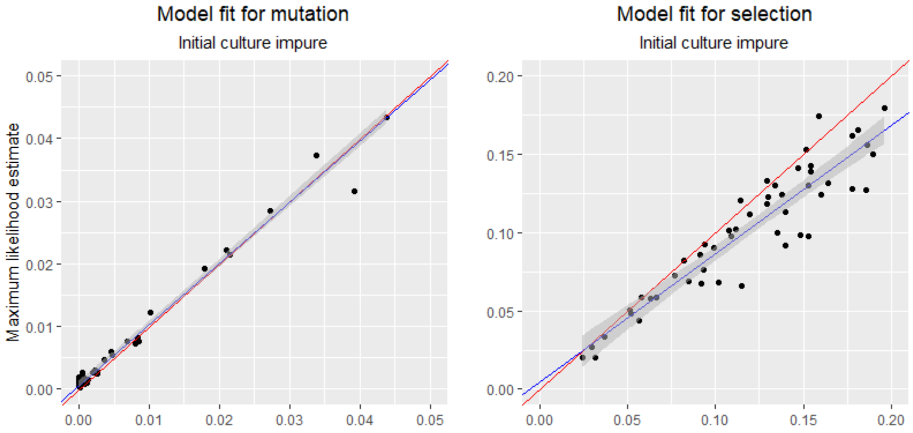

Figure S3: Comparisons of parameter estimates with true values from simulated data: effect of initially impure cultures. The starting culture used by the simulated data sets contained between 5-50 individuals carrying the transgene, thus at a frequency not usually detectable in a sample of 100 (frequencies 0.0005-0.005). There is little difference in performance when compared to the ideal case with a best fit equation of $\mu_{estimate} = 0.0008 + 0.98\mu_{true}$ for mutation and $s_{estimate} = 0.0049 + 0.82s_{true}$. The red line indicates the 1:1 fit expected if our method worked perfectly whereas the blue line and shaded region indicates the realized fit. The shaded region is the 95% confidence interval for the realized fit. Simulations otherwise follow those for text Fig. 3, using the bolded parameter values in Table 2.

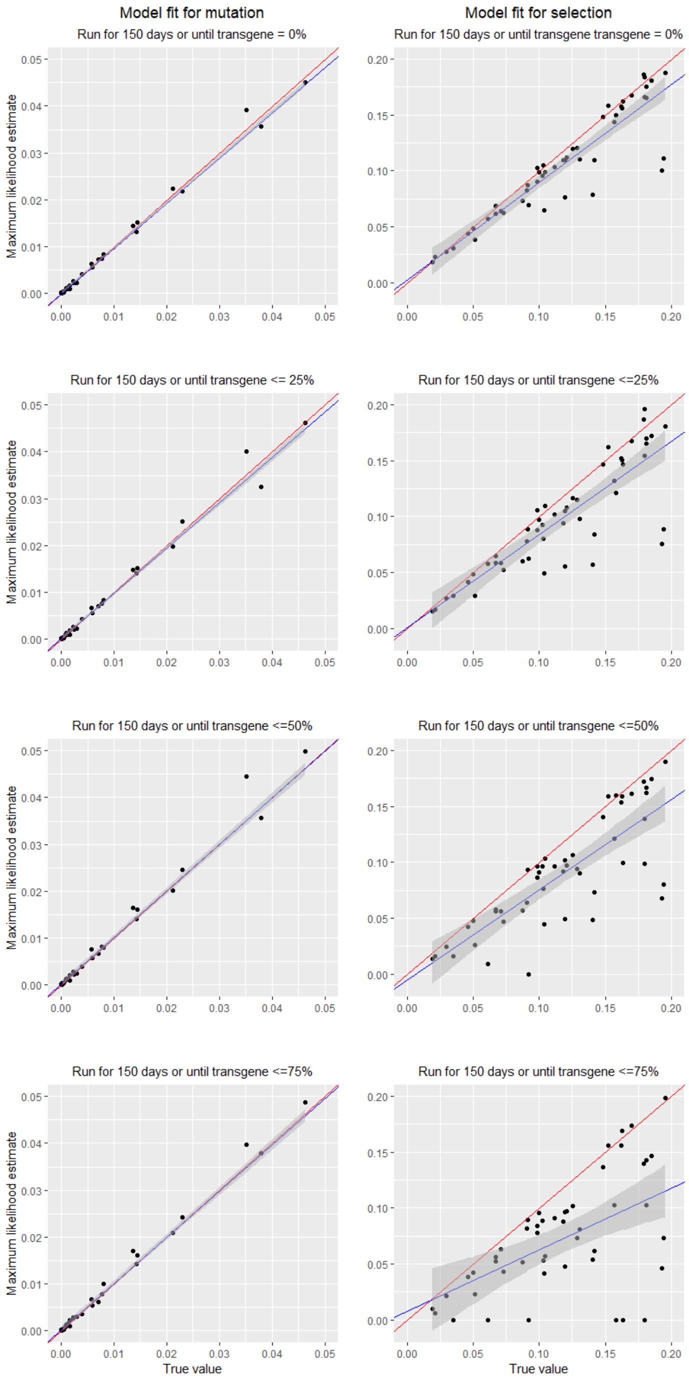
Model fit for mutation
Model fit for selection
Run for 150 days or until transgene = 0%
Run for 150 days or until transgene transgene = 0%
Run for 150 days or until transgene <= 25%
Run for 150 days or until transgene <=25%
Run for 150 days or until transgene <=50%
Run for 150 days or until transgene <=50%
Run for 150 days or until transgene <=75%
Run for 150 days or until transgene <=75%
Maximum likelihood estimate
True value

Figure S4: Comparisons of parameter estimates with true values from simulated data: effect of experimental duration. This figure addresses how long the serial transfer experiment must be carried out to achieve satisfactory estimates of mutation and selection. The measure of duration used here is based on how low the transgene frequency has declined. In the top row, the experiment is run for 150 days or until the transgene frequency reaches 0%. The next three rows down assume the serial transfers are terminated after 150 days or once the frequency of the transgene has dropped to 25%, 50%, and 75% respectively. Each panel assumes 3 replicates, 100 individuals tested for the presence of the transgene, a 24 hour transfer interval, initially pure culture, and the true growth rate value, r. The red line indicates the 1:1 fit expected if the method worked perfectly whereas the blue line indicates the realized fit. The shaded region is the 95% confidence interval for the realized fit. Best fit equations were $\mu_{estimate} = 0.0003 + 0.97\mu_{true}$ for mutation and $s_{estimate} = 0.0032 + 0.83s_{true}$ in the top row, $\mu_{estimate} = 0.0005 + 0.99\mu_{true}$ for mutation and $s_{estimate} = 0.0008 + 0.77s_{true}$ in the second row down, $\mu_{estimate} = 0.0008 + 0.85\mu_{true}$ for mutation and $s_{estimate} = 0.0044 + 0.74s_{true}$ in the third row down, $\mu_{estimate} = 0.0009 + 0.76\mu_{true}$ for mutation and $s_{estimate} = 0.001 + 0.68s_{true}$ in the bottom row. Simulations otherwise follow those for text Fig. 3, using the bolded parameter values in Table 2.

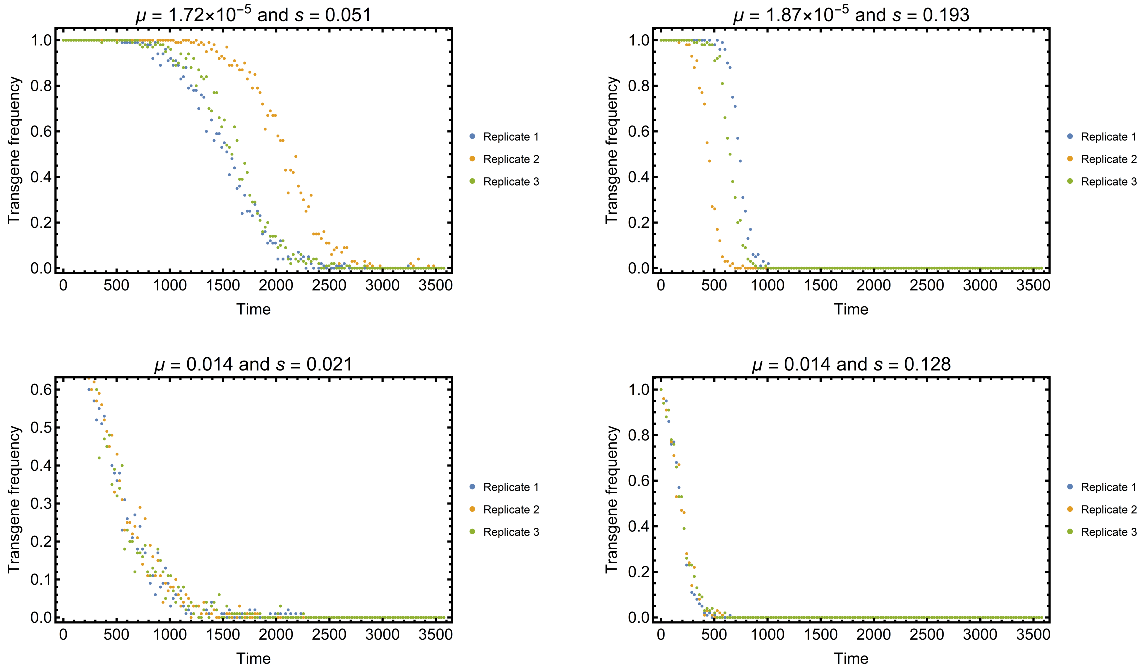

Figure S5: Plots of simulated transgene frequencies across transfers for each of three replicates. Each panel shows a different combination of selection and mutation and illustrates the variation in transgene frequency observed across replicate runs of the software used to test the fit of our maximum likelihood estimates to the true values. In all panels transfers were conducted every 24 hours and the presence or absence of the transgene was scored for 100 individuals at each transfer. The bottleneck size was 10,000. The carrying capacity was set to 2,000,000 and growth rate to 0.1 in all cases.

References

Hughes, J. M., Lohman, B. K., Deckert, G. E., Nichols, E. P., Settles, M., Abdo, Z., and Top, E. M. The role of clonal interference in the evolutionary dynamics of plasmid-host adaptation. *mBio*, 2012, **3**(4):e00077–00012. ISSN 2150-7511. doi: 10.1128/mBio.00077-12.
